# Leading public sector interorganizational collaboration in healthcare: Lessons from the intersection of climate and health

**DOI:** 10.1177/08404704241311911

**Published:** 2025-02-03

**Authors:** Amy Zidulka, Ingrid Kajzer Mitchell

**Affiliations:** 1 8202 Royal Roads University, Victoria, British Columbia, Canada.

## Abstract

It is generally accepted that wicked problems cannot be addressed by a single organization and require multiorganizational arrangements across governmental jurisdictions and sectoral boundaries. Health leaders increasingly are being called upon to lead collaborative initiatives. However, doing so is fraught with complexity. This article draws on relevant organizational literature and an empirical study focused on public sector collaboration for the purpose of fostering climate resilience in the health system to put forward four guidelines for collaborative leaders.

## Introduction

It is generally agreed that, in the face of wicked, intractable problems, collaborative approaches, in which managers work across departmental, organizational and sectoral boundaries are needed.^1-3^ Wicked problems are, by definition, those for which no single clear understanding exists of the problem itself and around which diverse stakeholders maintain different, often deeply held interests and values.^4^ Collaboration is appropriate for the purpose of deepening understanding of the challenge through tapping into the expertise held by different stakeholders and engaging in collective sense making.^5^ Collaboration can also assist in navigating the challenge through exploration of mutually beneficial solutions and development of trust and mutual commitment.^2^ In the realm of healthcare, the challenge of delivering quality care within an ever-more complex landscape – while also managing costs and addressing staffing issues – has been characterized as a wicked problem,^6,7^ as have more specific issues, including strategies for healthy aging, reducing hospital readmissions, substance abuse, and end-of-life care.^8,9^

However, the leadership literature has acknowledged that specialized competencies are required to engage in what has been termed integrative leadership, collaborative leadership, and boundary spanning leadership.^10^ Research also has identified interorganizational collaboration is easier said than done, and meaningful outcomes are extremely difficult to achieve.^1,9^ As explained by Williams:

“The context for collaboration is complex and challenging—typically characterized by an absence of clarity around purposes and problems; different value orientations, frames and systems of belief; unclear, multiple, shifting or conflicting goals; lack of precision around personal and organizational roles and responsibilities; differences in interpretation over what constitutes “success”; variations in culture and language; multiple accountabilities and performance management regimes; and conflicts between different modes of governance.”^11^

Interorganizational collaboration can be understood as both vitally important and fraught with difficulty.

This article draws on relevant organizational literature and empirical research to present guidelines for health leaders engaged in convening and maintaining interorganizational collaborations. The empirical research, sponsored by the British Columbia (BC) Ministry of Health, focused on collaboration in service of fostering health system resilience in the face of climate change. Initiated in 2022 the study involved qualitative interviews with relevant leaders from Canada, the Unites States, and Australia. This article points both to general guidelines, applicable to many healthcare situations, as well as illustrative examples emerging from our climate resilience research. After presenting relevant foundational literature and the study’s methodology, it offers guidelines for interorganizational collaboration.

## Foundations of interorganizational collaboration

Theoretically, interorganizational collaboration can be understood as comprised of five elements:^1,12^

**Figure 1.**
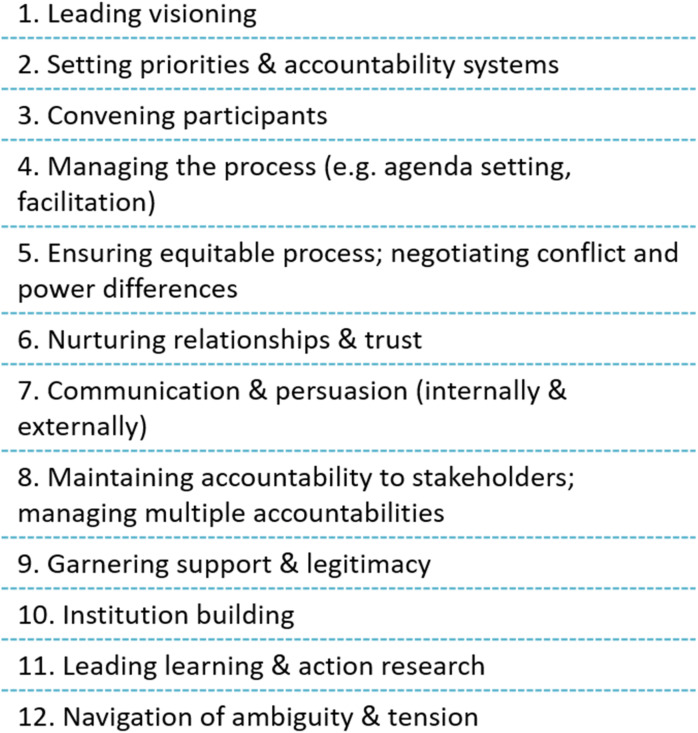
Leadership competencies for interorganizational collaboration.^5,23,24^

**1. Initial conditions** in which a collaboration arises, for instance, the degree of turbulence in the external environment and the strength of existing relationships between collaborating parties.
**2. Leadership**, which refers to the actions leaders take in facilitating collaboration, for instance, convening and running collaborative forums and building trust amongst key parties. Key leadership competencies are listed in Figure 1. Typically the following roles are needed:^13^ (1) sponsors, who possess formal authority to deploy resources and grant legitimacy; (2) champions, who convene, facilitate, and energize; (3) catalysts, who create a disturbance that activates others; and (4) implementers, who ensure execution.
**3. Institutional structures and governance systems,** for example, joint interorganizational networks and joint governance committees.
**4. Contingencies and constraints** that impact decisions around collaborative strategy, including collaboration type, power imbalances, and competing institutional logics.
**5. Outcomes** that can be expected, which determine the accountability systems that must be put in place.

These elements are related, with outcome expectations hinging upon the degree to which elements like the initial external environment conditions or governance structures are favourable to collaborative success.

### Collaborative capacity as an outcome

Understandably, leaders typically enter collaborations expecting tangible results, like improved policies, decreased emissions, or procurement of funding. However, when addressing wicked problems, achieving these results typically occurs in the long term and, moreover, it is unrealistic to expect the benefits of collaboration results when most health leaders operate in hierarchical bureaucratic and siloed systems^14^ that can actively work against interorganizational collaboration.

**Figure 2.**
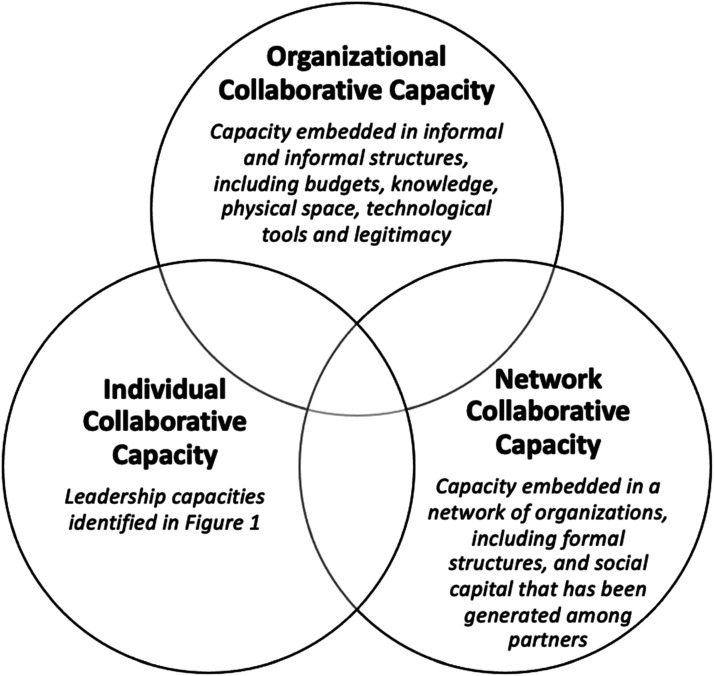
Three levels of collaborative capacity.^1,25,26^

It is therefore recommended that leaders consider the development of collaborative capacity, ‘the capability of organizations (or a set of organizations) to enter into, develop, and sustain interorganizational systems in pursuit of collective outcomes’,^15^ as a goal in itself whose achievement must precede and run parallel to the realization of tangible results. As illustrated in Figure 2, collaborative capacity requires attention at three levels – that of the individual, organization, and network. It is possible to document progress in collaborative capacity achievement through considering, for example, the quantity and quality of interorganizational relationships^7^ or the proficiency of leaders in known collaborative competencies.^16^

## Collaborating for a climate resilient health system

The potential impacts of climate change on human health can be considered a wicked problem whose consequences could include increased injury, disease, and death due to heat waves and fires and increased risks of food- and water-borne diseases.^17^ In response, the World Health Organization and other institutions^6,17^ have called for the development of climate resilient health systems, which can ‘anticipate, respond to, cope with, recover from and adapt to climate-related shocks and stress’,^17^ and whose realization requires ‘collaboration to develop a shared vision among diverse stakeholders, and coordinated cross-sectoral planning to ensure that policies are coherent and health promoting, particularly in sectors that have a strong influence on health, such as water and sanitation, nutrition, energy and urban planning’.^17^ Interorganizational collaboration therefore can be understood as essential to health system resilience in response to climate change.

While all interorganizational collaboration is challenging, working at the climate/health intersection is particularly so, since it calls on health leaders to work not only in familiar partnership constellations (e.g., between government bodies and medical professional groups) but also with new, unfamiliar collaborators.

It is within this context that we sought to identify guidelines for successful climate/health collaboration. Our year-long qualitative study, approved by our university’s research ethics board, was guided by the question, ‘What are the best practices in fostering interorganizational and cross-sectoral collaboration that can be learned from relevant national and international jurisdictions for the purpose of building the foundation of a climate resilient health system in BC?’ and involved interviews with 17 key informants from Canada, the United States, and Australia. The relatively small sample size reflects the qualitative nature of the study, which prioritizes in-depth exploration of participants’ experiences and perspectives over broad generalizability.^17,18^

Interviewees were purposively selected^19^ for their ability to provide relevant insight to those advancing climate resilience work within the BC Ministry of Health. We therefore prioritized interviewees engaged in interorganizational collaborative initiatives that: (1) offered inspirational examples of outcome achievement, (2) were convened by government, (3) were focused at the climate/health intersection, and (4) were positioned within systems structurally similar to the BC health system, with similar priorities including a commitment to collaborating with Indigenous partners. The limited number of initiatives that met all criteria necessitated inclusion of interviewees that met some but not all criteria, for instance, those involved in inspirational collaborations that were not government led or those involved in inspirational government-led partnerships with Indigenous groups focused on health but not climate. Interviews were semi-structured and sought to identify factors contributing to the successes and challenges experienced by interviewees.

We employed an interactive, emergent approach to data analysis, iteratively moving between the data and academic literature.^20^ Specifically, we began coding by using the five elements of interorganizational collaboration from the literature identified above (initial conditions, leadership, institutional structures and governance systems, contingencies and constraints, and outcomes), and then developed sub-codes by supplementing the academic literature with what emerged from interview data. This hybrid thematic analysis supported by NVivo software, balanced open-ended exploration with insights from pre-existing theoretical frameworks.^21^ Our study led to the identification of success factors relevant to collaboration in service of a climate resilient health system. In arriving at the four key recommendations highlighted in this article, we synthesized the dominant findings, with the first recommendation pointing to the import of initial conditions, the second to leadership, and the third and fourth to structures and governance systems.

## Recommendations for interorganizational collaboration

It is recommended that health leaders engaged in interorganizational collaboration work consider the following:

### Analyse initial conditions and align collaborative strategies and expected outcomes accordingly

Two levels of initial conditions should be considered: one, the local organizational and regional conditions, and two, the conditions established by broader societal norms.

Based on the empirical data, organizational and regional conditions identified as impacting the collaborative process and outcomes included: the degree of felt urgency around addressing climate change; the presence or absence of policy mandates; the support of political leaders and their willingness to make public commitments; the way in which the health system is organized, with less fragmented systems possessing increased collaborative capacity; the robustness of existing interorganizational relationships and networks; and, within the organization, the formal collaborative structures in place (e.g., joint committees across divisions), the resourcing allocated, the support of top leadership, and the presence staff who were personally passionate about the issues.

An illustrative example emerged from an interviewee from Victoria, Australia, where the Department of Health achieved successful collaborative results with internal governmental and external jurisdictional partners to put in place a robust climate change adaptation plan.^22^ Without diminishing the role strong leadership played in this collaborative success, it is significant that Victoria had experienced wildfires, that climate action was politically mandated, and that a Minister for Climate Action was in place. The broader environment thus enabled collaborative success.

However, most interviewees in our study were operating under quite different circumstances, with many facing daunting initial conditions for climate-focused collaboration. Climate resilience remains a relatively new area of focus within the broader sphere of healthcare and key documents used by study participants, for example, the 2020 Health Canada guide to conducting climate change and health vulnerability and adaptation assessments,^23^ are only a few years old. Consequently, most of those leading climate/health collaborations must engage in a process of trial and error, taking personal risks, acting entrepreneurially in connecting with a range of potential partners, in order to find some with whom they can establish common ground. Many interviewees spoke of significant time spent simply explaining the climate-health connection to stakeholders who had not previously considered the topic area. Clearly, if the initial collaborative conditions are such that potential partners do not even speak a common language, it is likely wise to aim for outcomes that speak to the development of collaborative capacity and defer the achievement of ambitious tangible results.

### Use multiple strategies for trust building

Nurturing relationships through getting to know partners personally over time is what often first comes to mind when considering trust building – and relationship building indeed is important. However, it is only one strategy. Trust should also be developed through establishing ‘procedural justice’, which refers to ensuring transparent, fair, and democratic decision-making processes^24^ and through the demonstration of outcomes.

Our study’s interviewees emphasized that interpersonal relationship building took significantly more time than was formally recognized. They spoke of strategies such as building white space into meetings to allow for emergent conversations. They also emphasized the particular importance of trust in climate/health collaborations, since these collaborations call on partners to show vulnerability in acknowledging what they do not know and, in the face of great uncertainty, to navigate and change course together – as opposed to simply committing to a fixed, pre-established plan.

However, trust in the process can be just as important as interpersonal trust. This was illustrated by an interviewee who successfully convened stakeholders who, in other forums, were adversaries, with some even facing each other in court. She credited the collaboration’s intricate and clearly outlined governance structure for enabling trust, describing it as the ‘secret sauce’ that enabled ‘democracy at its finest’. Importantly, collaborators quickly lose trust – and might withdraw from collaborative processes – when procedural justice is not apparent. A particularly common barrier to trust occurs when conveners convene stakeholders without specifying whether and how their input will inform decision-making, thus leading stakeholders to question whether they are wasting their time on an unfairly managed process.

The demonstration of outcomes is another key strategy for trust building, although admittedly it can be challenging if collaborations are at an early stage, and capacity is still developing. That said, outcomes can be process focused (e.g., a document outlining a co-created and shared purpose statement or theory of change) or small. Even ‘small wins’ can contribute in meaningful ways since ‘with each positive outcome, trust builds on itself incrementally, over time, in a virtuous circle’.^25^ An Ontario interviewee emphasized the importance of communicating outcomes in concise ways – particularly when working to gain the trust of senior executives – and spoke of the persuasive power of metrics and scorecards to report both on results achieved and on the health of the collaboration.

### Ensure adequate resourcing for internal staff and external partners

Collaboration is time consuming, and collaborative initiatives often unfairly rely on committed employees’ invisible labour that is not accounted for within formal Human Resource (HR) systems and thus is under-resourced. While reliance on employees overextending themselves may yield short-term results, it is not a reliable strategy for addressing wicked problems, which requires systemic approaches over the long term. This quote, from a participant in the United States, echoes the sentiment emphatically expressed by many of our interviewees:

“The cost of relationships and the value of relationships is deeply underinvested in. It is very expensive in people time and people power to build this level of trust…. If you’re going to commit to something you need to commit to the actual bandwidth that’s needed to do the labour.”

Equally important is ensuring the availability of resourcing for external partners. In particular those from smaller organizations and Indigenous and marginalized groups must be compensated for their time.

### Build Indigenous collaboration into the foundation

Several interviewees spoke to the importance of collaborating with Indigenous groups in working toward a climate resilient health system, emphasizing how, as one expressed, ‘there is so much to learn from and re-adopt from Indigenous knowledge and perspectives’. Indeed, the framework developed by the United States Centre for Disease Control to guide officials in preparing for the health effects of climate change has been adapted to reflect more holistic Indigenous perspectives ‘determined through generations of knowledge and practices developed through connection to lands and waters’.^26^

However, leaders are advised to eschew simply positioning Indigenous partners as sources of knowledge, which will then be applied within colonial structures. Rather, Indigenous collaboration should be built foundationally into governance structures. The most robust partnerships we studied had Indigenous representatives making decisions around collaborative principles, mission, objectives, and resource allocation from the very start. We were particularly inspired by interviewees from New South Wales, Australia, who embedded a shared governance model into a mainstream health unit so that Indigenous voices were structurally positioned at the heart of decision-making.^27^ These interviewees also emphasized how governments can better partner with Indigenous groups if Indigenous people are inside government, functioning as leaders and employees. Our findings emphasize the need to explore effective collaboration with Indigenous partners, specifically how leaders can address systemic power imbalances within existing colonial healthcare structures. This includes examining ways to enact governance models that prioritize power-sharing, co-designed decision-making and Indigenous leadership and self-determination.^28,29^ Clear frameworks are required to guide collaborative processes that meaningfully incorporate Indigenous knowledge into leadership practices, providing actionable steps for fostering equitable partnerships and embedding Indigenous ways of knowing into governance and decision-making.

## Conclusion

The primary focus of this article was to identify best practices in fostering interorganizational and cross-sectoral collaborations for the purpose of building the foundation of a climate resilient health system. We have highlighted how interorganizational collaboration, while vitally important for addressing many of our most pressing healthcare challenges, is challenging to navigate. Our findings point to the need to align collaborate strategies and outcomes with initial conditions, nurture trust building through multiple process and outcome-based strategies, provide adequate resourcing not only internally but also externally to increase collaborative capacity of key priority groups such as Indigenous and marginalized groups, and to build Indigenous perspectives and knowledge into the foundational governance structure from the onset of any interorganizational collaboration. While this study provides valuable insights into fostering collaboration for the purpose of building a climate resilient health system, certain limitations must be acknowledged. The relatively small sample size and scope, characteristic of qualitative research, mean that the findings are not intended to generalizable to all contexts. Instead, the study has aimed to provide an in-depth understanding of complexities and opportunities inherent in interorganizational collaborations for climate resilience in health systems, offering actionable insights for leaders navigating similar challenges. Our final recommendation, on fostering Indigenous collaboration, merits expansion that is beyond the scope of this article including broadening the lens of analysis to account for the broader colonial context in which healthcare operates and further linkage to existing frameworks for Indigenous-colonial collaboration. We hope this article’s guidelines will prompt leaders to avoid underestimation of potential collaborative challenges and provide a starting point for their mitigation.

## References

[1] Bryson JM Crosby BC Stone MM . Designing and implementing cross‐sector collaborations: needed *and* challenging. Publ Adm Rev. 2015;75(5):647-663. doi:10.1111/puar.12432.

[2] Head BW Alford J . Wicked problems: implications for public policy and management. Adm Soc. 2015;47(6):711-739. doi:10.1177/0095399713481601.

[3] O’Leary S Lieberman S Gulyas A , et al. Management actions to address the climate emergency: motivations and barriers for SMEs and other societal micro/meso-level groups. Int J Manag Educ. 2023;21(3):100831. doi:10.1016/j.ijme.2023.100831.

[4] Weber EP Khademian AM . Wicked problems, knowledge challenges, and collaborative capacity builders in network settings. Publ Adm Rev. 2008;68(2):334-349. doi:10.1111/j.1540-6210.2007.00866.x.

[5] Gray B Purdy J . Collaborating for Our Future, Vol. 1. Oxford: Oxford University Press; 2018. doi:10.1093/oso/9780198782841.001.0001.

[6] Holmes BJ Best A Davies H , et al. Mobilising knowledge in complex health systems: a call to action. Evid Policy. 2017;13(3):539-560. doi:10.1332/174426416X14712553750311.

[7] Thomas W Hujala A Laulainen S McMurray R , eds. The Management of Wicked Problems in Health and Social Care. 1st ed. New York: Routledge; 2018. doi:10.4324/9781315102597.

[8] Feo R Urry K Conroy T Kitson AL . Why reducing avoidable hospital readmissions is a ‘wicked’ problem for leaders: a qualitative exploration of nursing and allied health perceptions. J Adv Nurs. 2023;79(3):1031-1043. doi:10.1111/jan.15220. 35332579

[9] Huxham C Vangen S . Managing to Collaborate. New York: Routledge; 2013. doi:10.4324/9780203010167.

[10] Morse RS . Integrative public leadership: catalyzing collaboration to create public value. Leader Q. 2010;21(2):231-245. doi:10.1016/j.leaqua.2010.01.004.

[11] Williams P . The competent boundary spanner. Publ Adm. 2002;80(1):103-124. doi:10.1111/1467-9299.00296.

[12] Bryson JM Crosby BC Stone MM . The design and implementation of cross‐sector collaborations: propositions from the literature. Publ Adm Rev. 2006;66(s1):44-55. doi:10.1111/j.1540-6210.2006.00665.x.

[13] Crosby BC t Hart P Torfing J . Public value creation through collaborative innovation. Publ Manag Rev. 2017;19(5):655-669. doi:10.1080/14719037.2016.1192165.

[14] Liu G Tsasis P . A health system policy framework on “how to” build cross-sectoral collaboration: perspectives from health administrators and leaders. J Manag Policy Pract. 2024;25(1):94.

[15] Hoceva SP Jansen E Thomas GF . Inter-organizational collaboration: addressing the challenge. Homel Secur Aff. 2011;7(2). https://www.hsaj.org/resources/uploads/2022/05/7.2.5.pdf. Accessed December 9, 2024.

[16] Emerson K Smutko LS . UNCG Guide to Collaborative Competencies. Portland: Policy Consensus Initiative and University Network for Collaborative Governance; 2011.

[17] Merriam SB . Qualitative Research: A Guide to Design and Implementation. Los Angeles: Jossey-Bass; 2005.

[18] Creswell JW . Qualitative Inquiry and Research Design: Choosing Among Five Approaches. 3rd ed. Simi Valley. Sage Publications; 2013.

[19] Patton MQ . Qualitative Research & Evaluation Methods. 3rd ed. Thousand Oaks. Sage Publications; 2002.

[20] Nicolini D . Zooming in and out: studying practices by switching theoretical lenses and trailing connections. Organ Stud. 2009;30(12):1391-1418. doi:10.1177/0170840609349875.

[21] Fereday J Muir-Cochrane E . Demonstrating rigor using thematic analysis: a hybrid approach of inductive and deductive coding and theme development. Int J Qual Methods. 2006;5(1):80-92. doi:10.1177/160940690600500107.

[22] Victoria State Government . Health and human services climate change adaptation action plan 2022-26. 2022. https://www.dffh.vic.gov.au/publications/health-and-human-services-climate-change-adaptation-action-plan-2022-2026.

[23] Health Canada . Climate change and health vulnerability and adaptation assessments: resource guide. 2020. https://www.canada.ca/en/health-canada/services/publications/healthy-living/climate-health-adapt-vulnerability-adaptation-assessments-resource-guide.html. Accessed December 9, 2024.

[24] Kim J . Distributive justice in collaborative outputs: empowering minority viewpoints through deliberation. J Publ Adm Res Theor. 2024;34(1):1-15. doi:10.1093/jopart/muad012.

[25] Vangen S Huxham C . Nurturing collaborative relations: building trust in interorganizational collaboration. J Appl Behav Sci. 2003;39(1):5-31. doi:10.1177/0021886303039001001.

[26] Schramm PJ Ahmed M Siegel H , et al. Climate change and health: local solutions to local challenges. Curr Environ Health Rep. 2020;7(4):363-370. doi:10.1007/s40572-020-00294-1. 33113083 PMC7591693

[27] Crooks K Law C Taylor K , et al. Embedding aboriginal cultural governance, capacity, perspectives and leadership into a local public health unit incident command system during COVID-19 in New South Wales, Australia. BMJ Glob Health. 2023;8(7):e012709. doi:10.1136/bmjgh-2023-012709. PMC10357298 37460245

[28] Brugnach M Craps M Dewulf A . Including indigenous peoples in climate change mitigation: addressing issues of scale, knowledge and power. Clim Change. 2017;140(1):19-32. doi:10.1007/s10584-014-1280-3.

[29] Crowshoe LL Sehgal A Montesanti S , et al. The Indigenous primary health care and policy research network: guiding innovation within primary health care with Indigenous peoples in Alberta. Health Pol. 2021;125(6):725-731. doi:10.1016/j.healthpol.2021.02.007. 33685657

